# Association with humans and seasonality interact to reverse predictions for animal space use

**DOI:** 10.1186/s40462-018-0123-7

**Published:** 2018-04-28

**Authors:** Peter N. Laver, Kathleen A. Alexander

**Affiliations:** 1 Department of Biological Sciences, University of Cape Town, Private Bag X3, Rondebosch, 7701 South Africa; 2 Department of Fish and Wildlife Conservation, Virginia Tech, 310 West Campus Drive, Blacksburg, VA, 24061 USA; 3 Centre for African Resources: Animals Communities and Land Use (CARACAL), Lot 3102 Airport Road, Kasane, Botswana

**Keywords:** Home range, Resource dispersion hypothesis, Metabolic theory, *Mungos mungo*, Anthropogenic change

## Abstract

**Background:**

Variation in animal space use reflects fitness trade-offs associated with ecological constraints. Associated theories such as the metabolic theory of ecology and the resource dispersion hypothesis generate predictions about what drives variation in animal space use. But, metabolic theory is usually tested in macro-ecological studies and is seldom invoked explicitly in within-species studies. Full evaluation of the resource dispersion hypothesis requires testing in more species. Neither have been evaluated in the context of anthropogenic landscape change.

**Methods:**

In this study, we used data for banded mongooses (*Mungos mungo*) in northeastern Botswana, along a gradient of association with humans, to test for effects of space use drivers predicted by these theories. We used Bayesian parameter estimation and inference from linear models to test for seasonal differences in space use metrics and to model seasonal effects of space use drivers.

**Results:**

Results suggest that space use is strongly associated with variation in the level of overlap that mongoose groups have with humans. Seasonality influences this association, reversing seasonal space use predictions historically-accepted by ecologists. We found support for predictions of the metabolic theory when moderated by seasonality, by association with humans and by their interaction. Space use of mongooses living in association with humans was more concentrated in the dry season than the wet season, when historically-accepted ecological theory predicted more dispersed space use. Resource richness factors such as building density were associated with space use only during the dry season. We found negligible support for predictions of the resource dispersion hypothesis in general or for metabolic theory where seasonality and association with humans were not included. For mongooses living in association with humans, space use was not associated with patch dispersion or group size over both seasons.

**Conclusions:**

In our study, living in association with humans influenced space use patterns that diverged from historically-accepted predictions. There is growing need to explicitly incorporate human–animal interactions into ecological theory and research. Our results and methodology may contribute to understanding effects of anthropogenic landscape change on wildlife populations.

## Background

The Anthropocene is identified as a period of significant human influence on Earth’s ecosystems [1], although humans have been transforming large terrestrial areas and climate to some degree for millennia [2]. Given the global scale of anthropogenic impacts, a majority of free-ranging animals are likely already affected by anthropogenic landscape change [3]. During this period of change, free-ranging animals living in association with humans (“synanthropic” animals [4]) have been exposed to evolutionarily-novel costs and benefits associated with the increasing occurrence of anthropogenic resources, particularly in urban landscapes [5]. Much of our body of ecological theory, however, has been formulated from studies conducted on animals living without human association (“apoanthropic”) or under assumptions of negligible anthropogenic impact [6]. Yet, the increasing recognition of the transformative nature of human-induced landscape change is punctuated by the discovery of “novel” impacts on animal behavior and fitness [7]. Here, there is a need to reconsider how we perceive the benefits and costs of an animal’s habitat, a cognitive map [8], which may be based on historically-accepted theoretical models assuming little or no association with humans, and to reconsider how our study species interact with human-associated opportunities and costs. Without a possible re-calibration of our perceptions of animal resources and inclusion in relevant ecological theory we may develop biased inferences about our study systems, leading to suboptimal management and conservation outcomes.

For example, while animal space use in human-dominated landscapes has been investigated empirically, the associated theoretical framework has only received light treatment. For terrestrial species, however, movement and space use within home ranges is a fundamental component of their fitness. It defines where and how animals may satisfy metabolic requirements, find and use key resources, and find reproductive opportunities [9], but space use also imposes metabolic costs [10], opportunity costs [11], and exposes animals to conflict [12], competition [13], parasitism [14], and predation risk [15]. Metrics of space use represent trade-offs among these costs and benefits [16] and provide insight into animal ecology and fitness. In situations where free-ranging animals live in association with humans, these costs and benefits of space use may depart substantially from those observed in situations where free-ranging animals live without association with humans.

Many studies simply describe space use for a species through summary metrics developed from observation (e.g. area, number of individuals). But, these deterministic outcomes are only part of the general theory of ecology [17], in which variability in individual behavior and the environment are both important to the broader context and crucial in the evolutionary process. Understanding what drives this variation and resultant interactions is thus a key objective in ecological research. This focus on optimizing fitness through space use behavior forms a subset, the optimality paradigm, of a broader movement ecology paradigm [18].

Space use can be understood within the framework of the general theory of ecology, as adapted from [17] and [19]. Evolution results in general ecological properties of a species, for example, foraging or movement behaviors that result in a change in location and hence, a space use pattern. Such space use of animals with the highest fitness becomes characteristic for a species. Against this background, individual variability in space use among members of a species results in variable ecological patterns and processes. Further, environmental conditions and resource distributions vary in space and time. Foraging patterns then vary as a result of animals choosing among variable foraging options. Thus, animals move across a landscape unevenly in space and time. In their space use, animals interact with biotic and abiotic environmental variables, contributing to births and deaths. This animal fitness contributes in turn to the ongoing evolution of a species. Thus, within a species, space use, its variability, and the evolution of that species all depend to some degree on a) general characteristics of space use for the species, b) variability of space use among members of the species, c) variability of surrounding environments, and d) sensitivity to initial conditions at many spatial and temporal scales.

At macroecological scales, metabolic resources explain most space use variation, via allometric scaling rules [10, 13]. Although the scaling coefficients of the metabolic theory of ecology are controversial, the general theory predicts that large animals range farther than small animals to fulfill metabolic needs and that abundant resources allow high population densities and small home ranges [20]. Thus, behavior may be the primary method for preventing metabolic deficits, before physiological methods (e.g. glucocorticoid production) are used. In social animals, the resource dispersion hypothesis attempts to characterize cost-benefit relationships in the evolution of group living, and predicts a positive association between resource richness and group size, with home range size positively associated with resource dispersion [21]. The resource dispersion hypothesis is a sub-model of the resource productivity-variance model in which patch dispersion is used to model spatial variance in resources [22].

Drivers of variation of resource richness may include latitude and elevation, seasonality, meteorological variations (e.g. drought, rain), and anthropogenic landscape change [23, 24]. Critically, as human populations expand numerically and geographically, anthropogenic landscape change can displace, imperil, or extirpate a species [25]. But, these same human-mediated landscape changes may also modify resource availability, providing novel opportunities for species to live in association with humans [4].

For many species, the effects on ecology (e.g. group size, space use, and foraging ecology) of living in association with humans remain poorly understood. Considering the propositions inherent in metabolic theory, abundant anthropogenic resources allow constrained space use — which we term as “synanthropic metabolic theory”. This theory excludes potential effects of persecution by humans around anthropogenic resources. Yet, effects of living in association with humans appear species- and site-specific. Observational studies suggest negative [26], positive [27], and no association [28] between association with humans and home range size. Some species respond to anthropogenic resources by contracting core ranges but not home ranges [28] while others reduce several space use measures [26]. Different populations within species may also respond divergently e.g. [27, 29]. Experimentally, some species expand home ranges after losing anthropogenic resources [30], some maintain home range size during food supplementation [31], and some respond to clumped supplementation by increasing overlap but maintaining home range size [32].

Metabolic theory also predicts restricted space use during resource-plentiful seasons — which we term here “seasonal metabolic theory”. This theory excludes the effects of seasonal reproduction. Further, the predicted seasonal behavioral differences should result in similar metabolic outcomes between seasons. Effects of seasonal resource availability on space use may also vary from positive [33], to negative [34], to no association [35].

Research methodology may, however, affect home range inferences and account for disparate results within and among studies. For example, early metabolic theory studies used minimum convex polygon (MCP) home ranges, which are sensitive to sample size and outliers [36], include unused habitat, and depict only boundaries. Kernel density estimation (KDE) with asymptote analyses is less sensitive to sample size and outliers, excludes voids, and its utilization distributions reflect internal structure. Unfortunately, KDE core ranges are often delineated with arbitrary thresholds, usually 50% volume contours, which are potentially unrelated to space use concentration [37], and few studies follow or report steps for rigorous KDE [38]. Thus, study outcomes and inferences may vary due to analytical procedures employed, masking true ecological processes.

Here, we present home range KDE following [38] and test predictions from the space use theories introduced above using banded mongooses (*Mungos mungo*) in northeastern Botswana. Banded mongooses provide good models for space use studies. They are small-bodied (< 2 kg), diurnal herpestids exhibiting communal breeding [39] with limited social dominance [40, 41] and low reproductive skew [42, 43]. Within groups, banded mongooses generally den and forage together, allowing for tests of metabolic theory and the resource dispersion hypothesis. They also live readily in areas of anthropogenic landscape change, suffering little persecution, allowing for tests of effects of living in association with humans. For example, in Uganda, habituation to humans and access to anthropogenic waste affect banded mongoose space use, body condition, and demographics [28, 44]. In northeastern Botswana, banded mongooses also experience seasonality in precipitation and in the abundance and availability of their primary foods, soil macrofauna [45], allowing for tests of seasonality effects. Previous banded mongoose studies suggest inter-population variability in space use ecology, group size limits, and inter-group spacing (Table 1). Further, in Uganda, MCPs were positively associated with group size in one study [46], but not another that used KDE and MCP [28]. This variability among studies and across the geographic range of banded mongooses needs to be better understood, and our study adds to the existing information from the Serengeti [47], and Uganda. Using this empirical study system, we evaluate predictions and discuss implications for our theoretical understanding of space use and implications for future wildlife management and research.

**Table 1:** Banded mongoose population density, group size, and home range size in 7 ecosystems

Country	Site	Density^a^	Group size^b^^c^	n^d^	Home range^e^	n^d^	Study
Uganda	QENP^f^		(max = 32)				[87]
		17	14 (11 – 23)	6	^g^80 (38 – 130)	5	[45]
			14 (9 – 20)	14			[40]
			18 (9 – 27)	6			[42]
		16	12 (10 – 14)	10	76 (62 – 97)	10	[28]
		28		14			[88]
			^g^14 (3 – 36)	10			[89]
			^g^(8 – 44)	7			[90]
			16 (10 – 23)	8	^g^88 (30 – 132)	8	[91]
			^g^(7 – 44)	6			[92]
Tanzania	SNP^h^	0.5					[47]
		2	^g^15 (4 – 29)				[47]
Botswana	CNP^i^	8	13 (11 – 23)	35	68 (39 – 134)	10	This study
	Syn^j^		21 (10 – 27)	14	45 (37 – 98)	8	This study
	Apo^k^		13 (11 – 15)	21	131; 194	2	This study
Zimbabwe	HNP^l^		^g^(18 – 35)				[93]
South Africa	KNP^m^		(max = 75)				[94]
	MPNR^n^	4.6	18 (10 – 25)	8			[95]
	VCNR^o^	2.4					[96]

^a^km ^−2^

^b^Median (inter-quartile range) unless stated otherwise. Estimated from raw data where provided

^c^Studies may differ in including juveniles and sub-adults in counts, and in timing of counts

^d^Number of study groups

^e^Hectares (ha), median (inter-quartile range) unless otherwise stated

^f^Queen Elizabeth National Park: Savanna grassland

^g^Mean (range)

^h^Serengeti National Park: Short-grass plains; woodland

^i^Chobe National Park, Kasane, and Kazungula (overall): Woodland, riparian, urban

^j^Synanthropic groups only

^k^Apoanthropic groups only

^l^Hwange National Park

^m^Kruger National Park

^n^Mosdene Private Nature Reserve Woodland, floodplain

^o^Vernon Crookes Nature Reserve: Savanna grassland, forest

Following from the space use theories and previous studies introduced above, we tested 9 a priori predictions for banded mongooses. We based these predictions on the assumption that animals respond to changes in human-supplied foods in the same ways that they respond to such changes in natural foods. Our metrics of space use included home range sizes, core range sizes, day range distances, and the area-probability integral for dispersion of space use. We predicted under the simple metabolic theory that 1) there would be a negative association between soil macrofauna richness and space use, and that 2) group size and space use would be positively associated. Under seasonal metabolic theory, we predicted that 3) dry season space use would be more extensive than wet season space use while metabolic outcomes would be similar for both seasons. For the synanthropic metabolic theory we predicted that 4) mongoose groups without association with humans would have more extensive space use than mongoose groups living in association with humans, that anthropogenic resource richness — 5) measured by buildings or 6) measured by refuse (waste) sites — and space use would be negatively associated. The resource dispersion hypothesis predicted that 7) groups living in association with humans would contain more adults than groups living without association with humans, that 8) group size and space use would show no association, and 9) that patch dispersion and space use would be positively associated.

## Methods

### Study area and study animals

**Fig. 1:**
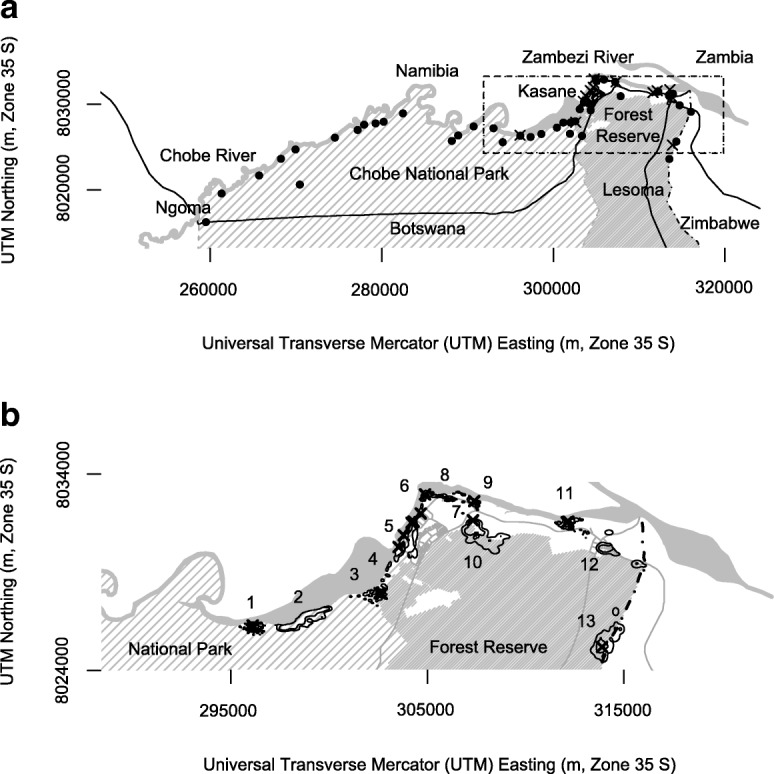
**a** Locations of 41 banded mongoose groups (black dots) along the Chobe River, northeastern Botswana (2008 – 2011). **b** Primary study groups (polygons of 95% kernel density home ranges, 1 to 13) lived in Chobe National Park (groups 1, 2, 3 and 4), Kasane Forest Reserve (groups 3, 10, 12 and 13), and the towns of Kasane and Kazungula (groups 5, 6, 7, 8, 9, 11 and 12). Groups living in association with humans lived at lodges (groups 1, 3, 5, 6, 7, 8, 9, 11 and 13), in towns (group 12) or in close association with a military camp (group 4). Two groups had no access to anthropogenic resources (groups 2 and 10). Black Xs indicate lodge or town refuse sites

We monitored 41 banded mongoose groups in northeastern Botswana from October 2007 to November 2011 (Fig. 1a), from which we obtained dry season group size estimates, based on the number of adults, for 35 groups. We focused our spatial analysis on 13 groups around Chobe National Park, Kasane, and Kazungula (Fig. 1b). The human population for this area was estimated to be 13 141 in 2011. From our mongoose study groups we obtained home range data for 10 groups, seasonal home range data for 8 groups, and day range data for 6 groups. Sample sizes, in this case, the number of groups, differ in our various analyses due to differences in access to groups and differences in levels of group tolerance of human observers. Access to groups differed by distance from our field station, variable road access in the national park, and national park gate opening and closure times, which prevented us from finding some groups while they denned. Detailed spatial analysis was only performed on groups for which we had reliable access, and for which our presence did not bias a group’s space use.

To assess food limitation using the proxy of fecal organic matter content, we collected and analyzed 1542 fecal samples from our 13 free-living mongoose groups over 138 sampling events from June 2008 to December 2010. From each study group we collected a median of 59 samples (range: 3 – 584) over a median of 6 sampling events (range: 1 – 54) from a median of 19 animals (range: 3 – 64). We also collected and analyzed 202 fecal samples from a captive control group during 68 sampling events from October 2008 to April 2011.

We housed 1 captive female and 3 captive male banded mongooses together in an outdoor enclosure (∼ 95 m^2^) at the CARACAL research facility in Kasane and fed them 820 g of canned wet pet food at 8 AM daily. We also supplemented the diet of these mongooses sporadically with natural food items such as coleopterans, spirostrepid millipedes, and bushveld rain frogs, *Breviceps adspersus*. These mongooses also foraged in their enclosure. While individuals were all fed together, consumption may have varied among individuals but we could not detect any dominance of the provisioned food resources by any individual in the group. These mongooses were raised in the facility from 2 weeks old and were 2 years old at the time of first sampling.

We classified groups as living in association with humans or living without association with humans by presence or absence of buildings within home ranges. Of the 10 groups for which detailed home range data were collected, 2 were characterized as groups with no access to anthropogenic resources (groups 2 and 10). To evaluate anthropogenic influences on space use, we delineated a scale of group association with humans using a singular value decomposition principal components analysis of 2 proxies of potential anthropogenic resources: tourist density as a proxy for food and building density for both food and denning opportunities. Dens were places where mongooses rested overnight and raised litters. Groups raised litters in maternity dens that they also used at other times for overnight rest, but there may have been characteristics of maternity dens that we were unaware of that precluded some overnight resting dens from being used as a maternity den. During the period between parturition and pup emergence, groups regularly moved from one maternity den to another. For the purposes of this study, we did not distinguish maternity dens from overnight resting dens. We estimated the density of buildings within a home range as the number of buildings digitized from satellite imagery divided by home range area. Density of buildings reflected both putative anthropogenic food waste and putative denning resources. Denning resources included (but were not limited to) building materials and scrap heaps (e.g. wooden planks, transport pallets), excavations under cement pathways and building foundations, French drains, septic tanks, drainage pipes and road culverts, and under floorboards inside buildings.

We log-transformed and standardized both the tourist and building densities by centering and scaling prior to decomposition using the *prcomp* function in the R *stats* package. The first principal component (PC1) explained 95% of the variability in the data and was used to describe levels of human association from more association (increasingly negative values) to less association (increasingly positive values). We transformed these values by multiplying by -1 so that the scale increases with increasing association with humans. We ranked the 10 groups in ascending order by PC1: from groups living without association with humans in either the Chobe National Park or the Kasane Forest Reserve (2 groups), to groups living predominantly at tourist lodges surrounded by either national park or forest reserve (3 groups, 1 lodge for each group), to groups living predominantly at tourist lodges and surrounding residential and commercial areas in the towns of Kasane and Kazungula (5 groups, with each group using a range from 1 to 6 lodges).

An unplanned before-after-control-intervention experiment occurred during our study, allowing us to observe banded mongoose behavior before and after one of the lodges closed its refuse site. This affected 1 mongoose group (ID = 1) which lived predominantly at this lodge within the Chobe National Park. This mongoose group had no access to other lodges but still had access to anthropogenic denning resources at this lodge and still raided the lodge kitchen refuse bins opportunistically when the kitchen door was left open. Access to refuse sites for our other study groups remained constant, and we similarly observed banded mongoose behavior in these “control” groups over the same unplanned intervention period.

Mongoose study groups occurred in riparian and adjacent *Baikiaea plurijuga*-dominated woodland, with an annual mean (SD) rainfall for the years 1994 to 2006 of 552 mm (148 mm). Rainfall was recorded at a meteorological station at the Kasane Airport, a site central to our study area, and rainfall data were supplied by the Republic of Botswana Department of Meteorological Services. Rainfall over the majority of the study period from 2008 to 2011 was slightly higher and more variable than the preceding decade, with a mean (SD) of 730 mm (224 mm) per year. At the beginning of the study, we used historic rainfall data to delineate a priori seasonal designations in the study design as wet, dry, or transition months. For the wet season we used a monthly delineation of mean > 50 mm, which occurred from November to March. For the dry season we used mean < 5 mm, which occurred from May to September. For the transition season we used 5 mm ≤ mean ≤ 50 mm, which occurred in April and October. Actual monthly rainfall approximately matched our a priori seasonal delineation, which we then retained for data analysis. The actual mean monthly rainfall (mm) for January to December, from 2008 to 2011 was 246, 96, 115, 41, 7, 11, 0, 0, 0, 6, 84, 123.

### Food resource richness and patch dispersion

We indexed resource richness using soil macrofauna and buildings. In our study area, deficiencies in public access to refuse disposal or collection result in the open disposal of anthropogenic food and non-food waste around residential and commercial buildings. Tourist lodges throughout the study site concentrated refuse at central non-animal-proof refuse sites on their properties before periodic removal to the Kasane landfill. We scaled the density of tourists within each mongoose group’s home range using the number of bed nights sold at lodges within the home range divided by home range area. This scaling reflected the anthropogenic food waste available at lodge refuse sites generated by guests eating at each lodge. During our study, no mongoose groups used the fenced Kasane landfill.

We digitized buildings, habitat types, and tree canopies from satellite imagery (Google Earth, Mountain View, CA, USA), verified with field observations. We counted the total number of buildings in each home range and quantified building density as the count divided by the home range area. We estimated available soil macrofauna in each group’s home range for each season using previously-published data for our study site [48] of habitat-specific macrofauna densities sampled to 20 cm depth, which is a typical foraging depth for banded mongooses. Thus, we calculated the area for each habitat in each mongoose group’s home range (overall and for wet and dry seasons) and multiplied those areas by the associated published macrofauna densities [48]. We then added the macrofauna totals over all habitats to yield an overall macrofauna count for each home range. This count, divided by area provided macrofauna densities (m^−2^) for each home range.

Soil macrofauna availabilities in various habitats were generally higher in the wet season than the dry season [48]. Macrofauna densities in closed canopy riparian areas increased 0.9-fold from 288 in the dry season to 549 in the wet season but decreased in open canopy riparian areas by 0.34-fold from 143 to 94. In *Baikiaea plurijuga* closed canopy areas, macrofauna increased 1.48-fold from 212 to 526 and increased in open canopy areas by 9.81-fold from 106 to 1146. In *Combretum*-dominated shrubland, macrofauna increased 1.11-fold from 140 to 296. In contrast, overall daily tourist occupancy across the study area increased 0.5-fold from wet season (347) to dry season (513), suggesting associated increases in anthropogenic food waste in the dry season.

Foraging patches may also be used because they provide cover from predators. Avian predators are responsible for most predation of banded mongooses, including martial eagles (*Polemaetus bellicosus*) in the Serengeti [49] and marabou storks (*Leptoptilos crumeniferus*, 50% of known mortalities) in Uganda [44]. Banded mongooses in Uganda also mob fish eagles (*Haliaeetus vocifer*) [49]. Other depredations in Uganda are attributable equally to reptilian predators, mammalian carnivores, warthogs (*Phacochoerus africanus*), and humans [44]. Of 55 adult mortalities from known natural causes in 2008 and 2009 in our study population, raptors (7.3%) caused 4 and mammalian carnivores 1. Other causes of mortality were due to disease or were urban-associated, with mortality due to *Mycobacterium mungi* infection causing 25, humans 20 (including roadkill), and domestic dogs 5. It is unclear from our study how living in association with humans may have altered banded mongoose perceived predation risk.

Delineating foraging patches is necessary for testing the resource dispersion hypothesis. But, patches may be difficult to delineate, and food resources may exist along a continuum or in diffuse patches in the environment [50]. How patches are delineated could affect a study’s results, but engaging *a posteriori* definitions of patches could lead to “fishing” for patch definitions that might provide the necessary support for the ecological theory under evaluation. *A priori*, we delineated foraging patches using tree canopy and building coverage because: 1) wet season canopy cover regulated soil moisture between rainfalls, increasing vertical migration of macrofauna, and closed canopy habitat had more invertebrates than open canopy habitat [48]; 2) trees, hollow logs, and buildings provided den sites and predation and temperature refugia for mongooses. We estimated patch dispersion using mean nearest neighbor distances [51, 52]. Thus we measured the mean distance from each tree or building (a patch) to its nearest neighboring patch for each mongoose group’s home range. Small mean nearest neighbor distances indicated high aggregation of patches, while large distances indicated low dispersion of patches. The relationship between patch dispersion and level of association with humans was uncertain as there were only 7:1 odds on groups exhibiting greater patch dispersion if they had greater levels of association with humans.

### Food limitation — fecal organic matter

Assessing food limitation in free-living animals poses significant challenges. Fecal dry matter comprises organic and inorganic matter. The latter, “total ash”, could originate from ingested substrate (e.g. soil) or diet. Soil ingestion during food limitation has been demonstrated in several species [53–61]. We indexed food limitation for mongoose groups using the median percentage fecal organic matter for each group (overall, and by season) and assumed that high organic matter content reflected low food limitation or high food availability. We determined sample organic content by ashing dried samples in a muffle oven [62].

As a proxy, fecal acid-insoluble ash may be a reliable marker of soil ingestion [60] and among 28 wildlife species assessed [63], fecal ash correlates positively and strongly with ingested soil. Total ash may provide an equivalent marker. We estimated total ash and acid-insoluble ash in a subset of 30 of our samples and found a strong positive relationship between the measures (Pearson’s *r*=0.94). Dietary ash may confound both markers, but invertebrates generally have high digestibility (78%) and low total ash content (5%) [64], excluding earthworms, geophagous termite workers, and termite soldiers [65]. For earthworms, soil may constitute 20 to 30% of dry weight [60]. But, dry savannas lack earthworms and none were found during invertebrate sampling in our study area [48] or recorded in banded mongoose diets in Uganda [45]. Ash content is low for typical banded mongoose prey items such as termite alates (7%) [66], and total ash of food fed to captive mongooses was 7 to 9%, whereas, ash content of mineral soil is generally > 90 % [63]. We thus assumed that fecal ash, or its inverse, fecal organic matter, is a good marker for soil ingestion and food limitation in our study: high fecal organic matter should reflect low food limitation.

### Telemetry

We telemetry-collared 36 mongooses in 13 groups but obtained sufficient space use data for home range analysis in only 10 groups. We kept collars on mongooses for a median of 158 days each (interquartile range [IQR] = 67 to 338). We trapped mongooses in rigid Tomahawk live traps (81.3 cm × 25.4 cm × 30.5 cm; Tomahawk Inc., Hazelhurst, Wisconsin, USA) baited with chicken or canned dog food. We telemetry-collared animals opportunistically when groups were without collared animals and, thereafter, only when collars needed replacement. Telemetry-collaring occurred mostly in the dry season (50%), followed by the wet season (26%) and transition months (24%). We avoided collaring while groups had pups in dens, a period which lasted for the month after parturition. We placed traps at locations that we knew groups visited on a reliable basis, or outside of dens when we knew the denning location from the previous evening. We immobilized mongooses using medetomidine hydrochloride (Domitor, Pfizer Inc., New York, NY, USA) at doses of 1.0 mg kg ^−1^, reversing anesthesia with atipamezole hydrochloride at the same doses (Antisedan, Pfizer Inc., New York, NY, USA). We replaced or removed collars as battery power dissipated. We conducted this study under permit from Botswana’s Ministry of Environment, Wildlife, and Tourism, with approval of Virginia Tech’s Institutional Animal Care and Use Committee (7-146-FIW).

We used 22 very high frequency (VHF) transmitters, and 4 global positioning system (GPS) transmitters, re-using transmitters on other animals in a few cases when collared animals were predated or when transmitters were dropped and those transmitters still had sufficient battery life. We collared adults (20% female; 80% male), selecting animals based on size without regard to sex. Large animals have preferable, low collar-to-body-mass ratios. Collared animals had a median mass of 1341 g (IQR: 1280 to 1515) and collars were 3% (median) of body mass (IQR: 2.1 to 3.4). We located groups by telemetry homing, approaching by foot or vehicle. Through homing, we could observe a collared animal and determine if it was with its group, or on a lone foraging foray (this never occurred), or in a den with pups prior to their emergence (this seldom occurred, and we found the rest of the foraging group within a few minutes in these cases).

One observer visited multiple groups daily, briefly recording location at first sighting, group size (adults), habitat, and behavior. This observer reduced potential bias from being present by using location at first sighting, and then remaining with groups only briefly, and visiting multiple groups daily. This observer used temporally-stratified sampling, searching extensively for VHF signals. Groups eluded this observer on 122 attempts (0.6% of our data) due to topography, collar malfunction, or mongoose behavior (e.g. denning). A second observer also found groups using telemetry homing and followed 2 groups per week for 24 h, recording group locations (31 locations daily, median). Again, we used temporally-stratified sampling among all groups at the scale of weeks, to reduce potential for spatial or temporal bias in data. If mongooses were perceived to move in response to this observer, the observer retreated from the group (infrequent occurrence, B. Fairbanks, personal communication). To minimize positional error from observations, we estimated group center and distance between 2 animals with farthest linear separation (median 15 m [IQR: 5 to 25]). We collected hand-held GPS fixes once groups departed. GPS collars attempted fixes once daily on randomized schedules during daylight, and once hourly for 10 h every 10 days.

We found dens of telemetry-collared groups by telemetry homing (or dens of other groups opportunistically) before mongooses emerged at dawn or after they retired at sunset on 525 nights (1239 den observations) for 17 groups. Most observations were from 10 telemetry-collared groups (a median of 126 observations per group [IQR: 28 to 173]). We also recorded foraging in refuse or drinking from anthropogenic water sources.

We counted adult group sizes by direct observation in open space. We classified animals as juvenile (∼ 0 to 6 months), sub-adult (∼ 6 to 12 months) or adult (approx. > 12 months) using body size, following cohorts from den emergence (approx. 4 weeks old) to calibrate size estimates. Our initial size-age classifications were developed between 2000 and 2007 using 5 mongoose groups. We obtained counts at almost every group observation. For the 10 groups for which we obtained home range data, for each month, we used the modal (most common) group size count for the monthly group size. Mongooses evicted from a group would occasionally spend time with other groups, and not all mongooses in a group could be counted at every observation. The modal count thus provided an estimate of the most consistent group size. For each season, we used the median monthly group size as the seasonal group size. For the remaining 25 groups for which we obtained dry season group size estimates, we used the median adult count obtained from opportunistic observation during the 4 dry seasons of the study.

### Home range estimation

We tested yearly site fidelity [67]. We assessed time to statistical independence [68] for 6 groups (17 357 fixes total, and a median of 2134 per group). For each group we determined hourly intervals with Schoener’s Ratio consistently > 2 and the interval where median Schoener’s Ratio for the 6 groups was > 2. We estimated displacement, the Euclidean distance on the 2-dimensional Euclidean plane defined by Universal Transverse Mercator map projection coordinates, from a group’s first sighting of the study to all subsequent sightings, pooling by group type. We estimated day range, as the daily distance traveled, determined by the Euclidean distance between consecutive fixes, for groups with ≥ 10 fixes spanning ≥ 5 h in a day, using 6 groups and 8993 fixes over 197 days. For our day range analyses, we had a median of 27 days per group, and a median of 38 fixes over a median of 8.5 h per day. We assessed asymptotes using area-observation plots [69], randomizing and resampling fixes for 11 groups, with 5 simulations each. We delineated asymptotes where 95% confidence intervals of simulations consistently fell within 15% of final home range size.

We estimated home range size for 10 groups (7093 fixes, a median of 589 fixes per group [IQR: 254 to 861]), using KDE with fixed, bi-weight kernels [70], volume contouring, and unit variance standardization [71]. We selected least-squares cross-validation (LSCV) bandwidths, which never failed, finding global minima for loss functions with golden section searches [72]. We applied a constant, *A*(*K*)=2.04, converting bandwidths from normal kernels (LSCV) for subsequent use with bi-weight kernels. We used grid resolutions of 75 cells along the shorter of *X* or *Y* axes. We delineated home ranges at arbitrary 95% volume contours, clipping contours to dry land. Thus, we excluded a water-filled quarry, water-retention dams, and the Chobe River, where 95% contours overlapped these features.

We estimated core ranges using area-probability curves [37]. We adapted these curves to estimate space use dispersion, computing definite integrals, $\int ^{100}_{0}f\left (x\right) dx$, with *f*(*x*) a plot of percentage home range as a function of probability of use, scaled by maximum probability of use. Smaller area-probability integrals (APIs) indicate use of smaller home range proportions for given probabilities of use (integrated over all probabilities) and hence, more concentrated space use. We estimated home ranges and core ranges over the study period and seasonal home ranges and seasonal core ranges for wet and dry seasons that excluded transition months. For home range, core range, and asymptote analyses, we used ABODE (Beta v. 5) [72] in ArcMap 9.3.1 (ESRI, Redlands, California, USA). For other analyses we used R [73]. We monitored access to lodge refuse sites for 8 groups over 4 years, and estimated yearly change in dry season ranges and dry season core ranges relative to starting sizes (i.e. in the first dry season of the study).

### Modeling drivers of space use

We modeled the effect of level of association with humans, measured using our singular value decomposition of tourist density and building density, on space use metrics (overall home range sizes, core range sizes, and area-probability integrals) and group size using Bayesian simple linear regression in R and STAN [74]. We used diffuse priors for intercepts (Cauchy[ *x*_0_=0, *γ*=10]) and slopes (Cauchy[ *x*_0_=0, *γ*=2.5]). We used 3 chains, with 1×10^3^ step burn-ins, and final Markov Chain Monte Carlo (MCMC) samples of 3×10^5^ iterations.

We tested for seasonal differences in space use metrics and resources within groups that had association with humans using Bayesian parameter estimation [75] in R and JAGS [76], modeling paired difference scores for estimates of day range, seasonal home range, seasonal core range, area-probability integral, building density, soil macrofauna density, and group size. We used diffuse priors modeled with *t* distributions centered on means of paired difference scores, with variance 10^6^-fold greater than score variance [75]. We assessed sensitivity to priors using skeptical priors (*t*[mean = 0, variance = 1]), which did not alter inferences. We used 3×10^3^ step burn-ins and 3×10^5^ iterations for final MCMC samples. We assessed seasonal differences in space use metrics qualitatively for the one group living without human association for which we had sufficient data.

We modeled the effects of putative space use drivers by season on 3 space use metrics, the area-probability integral (dispersion of space use), home range size, and core range size. Our putative space use drivers were buildings, macrofauna, patch dispersion, and group size, all detailed above. The area-probability integral is dimensionless and we modeled both total building or macrofauna count in a seasonal home range, and building or macrofauna density. For the home range size and core range size analyses, total building or macrofauna count would be conflated with the home range size or core range size, so we included only building or macrofauna density. For these analyses, we used Bayesian simple linear regression in R and STAN [74], using diffuse priors for intercept and slope (Normal [mean = 0, variance =1×10^6^]), and diffuse priors for variance of residual error (Cauchy[ *x*_0_=0, *γ*=5]). We used 3 chains, with 1×10^3^ step burn-ins, and final Markov Chain Monte Carlo (MCMC) samples of 3×10^5^ iterations.

For all Bayesian analyses, we assessed posterior predictive distributions graphically and with Bayesian *p* values to assess model fit. All models exhibited good fit in posterior predictive checks. We assessed MCMC chain convergence using trace-plot mixing, autocorrelation plots, and potential scale reduction factors, and we used 95% highest posterior density intervals (HPDI) for credible intervals. To aid interpretation, we summarized our updated belief in posterior distributions using subjective certainty bands: high certainty, where *β*=0 intersects < 5% of posterior distribution, moderate certainty, where *β*=0 intersects ≥ 5 to < 10% of posterior distribution, or uncertainty, where *β*=0 intersects ≥ 10% of posterior distribution. For example, an effect with 95% of the posterior > 0 (high certainty), has 19:1 or better odds for being a positive effect. Similarly, the odds for a positive effect described by the other bands would be between 19:1 and 9:1 (moderate certainty), and worse than 9:1 (uncertain). Ultimate interpretation of the weight of evidence rests with the reader and should rely on interpreting all the information contained in the posterior.

## Results

### Effects of association with humans on space use and group size

**Fig. 2:**
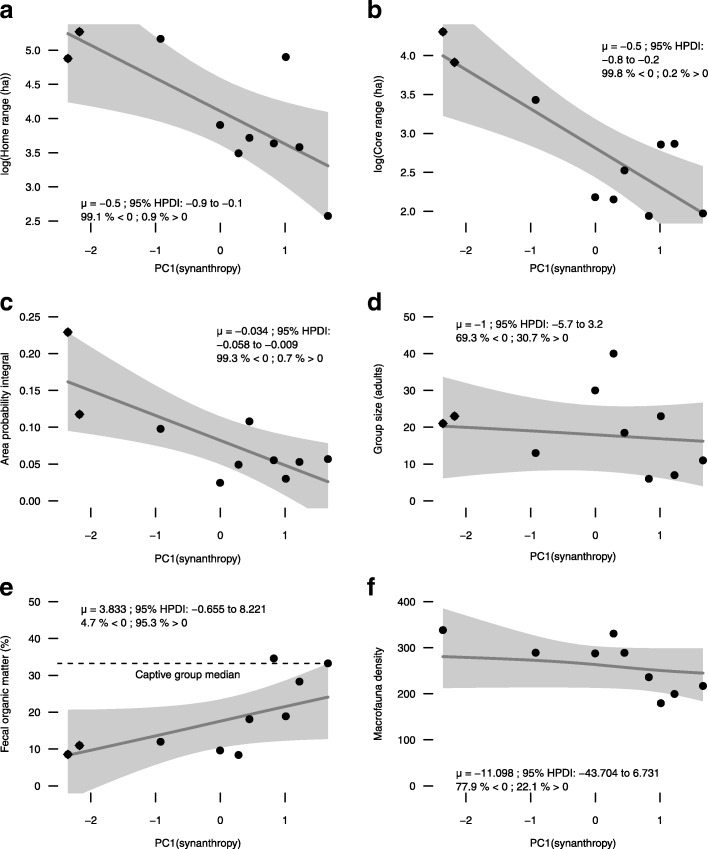
Banded mongoose home range (**a**), core range (**b**), space use dispersion (area-probability integral) (**c**), group size (**d**), fecal organic matter (**e**), and estimated macrofauna density (**f**) as functions of increasing association with humans (principal component 1, combining building and tourist density) in northeastern Botswana (2008 – 2011). The 2 groups lacking association with humans are depicted with diamonds. Bayesian posteriors on the slopes and their 95% highest posterior density intervals are summarized by the black lines with gray shading, respectively, and by the associated summary values. The median fecal organic matter content (%) for a captive group is depicted with a dashed line (**e**)

Banded mongoose space use was strongly related to a group’s level of association with humans, but group size was not. Further, a clear dichotomy existed between the space use of groups living in association with humans and groups living without association with humans. Among all groups, we had high certainty (> 110:1 odds) that there was a negative relationship between the level of association with humans (principal component 1) and overall home range size, overall core range size, and overall space use dispersion (Fig. 2a, b, c). But, there was an uncertain relationship between group size and level of association with humans (2:1 odds on a negative relationship between group size and level of association with humans) (Fig. 2d). We had high certainty (20:1 odds) that there was a positive relationship between the level of association with humans and the median fecal organic matter content for a mongoose group (Fig. 2e). The groups with the highest levels of association with humans had fecal organic matter content levels similar to those of a captive group that was fed canned pet food (Fig. 2e). The relationship between dry season soil macrofauna density and level of association with humans was uncertain (3.5:1 odds on a negative relationship between dry season soil macrofauna densities and level of association with humans) (Fig. 2f). The wet season soil macrofauna densities (not shown) also lacked a clear relationship with level of association with humans (1.6:1 odds on a negative relationship between wet season soil macrofauna densities and level of association with humans).

**Fig. 3:**
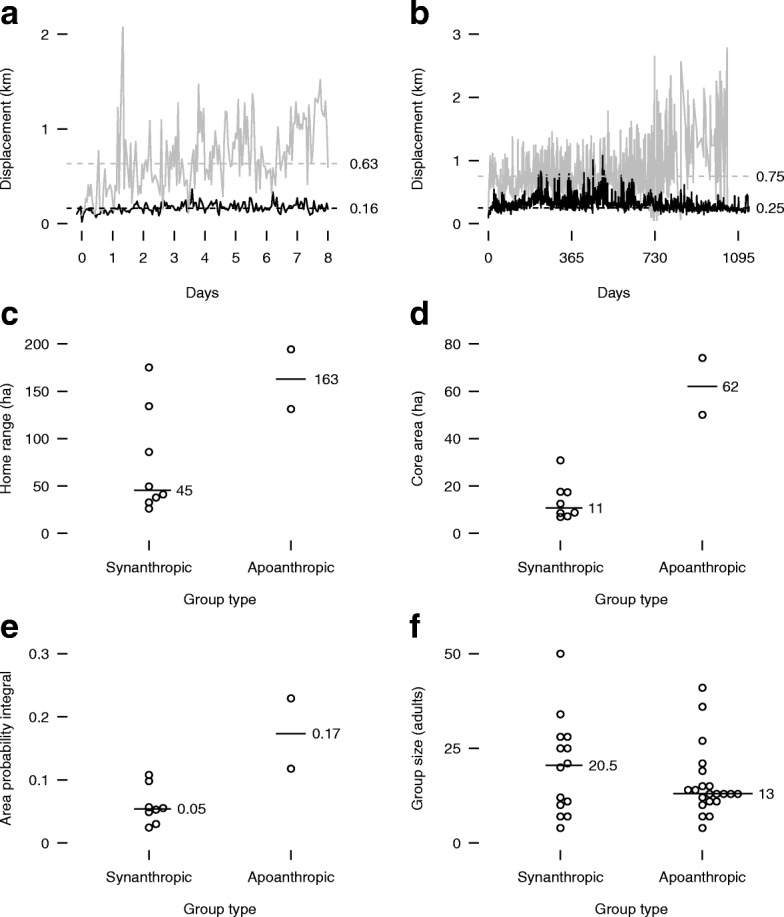
Banded mongoose median daily (**a**), hourly (**b**) displacement, home range (**c**), core range (**d**), space use dispersion (area-probability integral) (**e**), and group size (**f**) for groups living in association with humans (black lines) and groups living without association with humans (gray lines) in northeastern Botswana (2008 – 2011). Horizontal lines with associated values represent medians

When we separated the broad banded mongoose group categories to assess ecological effect sizes, groups that lacked association with humans had more dispersed space use with large effect sizes (Table 2, prediction 4), as exhibited by larger hourly (2.9-fold larger, Fig. 3a) and daily (2-fold larger, Fig. 3b) displacement (Euclidean distance from a group’s first sighting of the study to all subsequent sightings), larger home ranges (3.1-fold larger, Fig. 3c), larger core ranges (4.6-fold larger, Fig. 3d), and more space use dispersion (2.4-fold more dispersed, Fig. 3e). Under this dichotomy, Bayesian parameter estimation demonstrated no clear difference in group size between the 14 groups associated with humans and the 21 groups living without association with humans (95% HPDI: -0.3 to 0.7; posterior distribution: 21.8% < 0< 78.2%) (Fig. 3f, Table 2, prediction 7). Median dry season group size for all groups combined was 13 adults (IQR: 11 to 23; range: 4 to 50). The total dry season adult population for 35 groups we could reliably count across the study site was 597.

**Table 2:** Predictions and results concerning banded mongoose group size and space use relevant to the metabolic theory (including seasonality and association with humans) and the resource dispersion hypothesis

Theory	Metrics	Prediction or association	Result	Figure	Table	Outcome
Simple metabolic theory						
	1) Soil macrofauna richness and space use^a^					
		Negative	Positive (dry)		6; 7	Reversed
	2) Group size and space use^a^					
		Positive	Uncertain		6; 7	Not upheld
Seasonal metabolic theory						
	3) Space use^b^					
		Syn^c^: Dry > Wet	Dry < Wet	4	5; 4	Reversed
		Apo^d^: Dry > Wet	Dry > Wet	4	5; 4	Anecdotal
Synanthropic metabolic theory						
	4) Space use^e^					
		Syn^c^ < Apo^d^	Syn^c^ < Apo^d^	2; 3		Upheld
	5) Anthropogenic resource richness (buildings) and space use^a^					
		Negative	Negative (dry)		6; 7	Upheld
	6) Anthropogenic resource richness (refuse sites) and space use^f^					
		Negative	Negative			Anecdotal
Resource dispersion hypothesis						
	7) Group size					
		Syn^c^ > Apo^d^	Uncertain	2; 3		Not upheld
	8) Group size and space use^a^					
		None	Uncertain		6; 7	Upheld
	9) Patch dispersion and space use^a^					
		Positive	Uncertain		6; 7	Not upheld

^a^Seasonal space use dispersion (area-probability integral), seasonal home range size, seasonal core range size

^b^Seasonal day range, home range, core range, space use dispersion

^c^Synanthropic (living in association with humans)

^d^Apoanthropic (living without association with humans)

^e^Overall home range, core range, and space use dispersion

^f^Dry season home range size and core range size

### Effects of association with humans on behavior

Anthropogenic resources provided both denning and foraging opportunities and were used frequently by banded mongooses in the study site. We did not, however, determine the relative use, availability, and hence preference for these resources in our study. Groups used a median of 30 unique den sites (IQR: 27 to 36, *n*=10 groups), spending 2 to 3 consecutive nights at a particular den, and returning to previous dens after a median of 106 nights (IQR: 50 to 131, *n*=10 groups). Groups living in association with humans denned in man-made structures on 81% of nights (Table 3, *n*=11 groups). Groups living in association with humans fed from refuse in 110 of 850 (13%, n = 11 groups) foraging observations and drank from anthropogenic water sources in 78% of drinking observations, mostly from gray-water and sewage (21%) and lawn sprinklers (17%). We only observed mongooses drinking in 37 observations throughout our study.

**Table 3:** Percentage of nights spent in various den types by banded mongooses in northeastern Botswana (2008 – 2011) (this study) and Queen Elizabeth National Park, Uganda [45]. We used data for 11 groups living in association with humans and 6 groups living without association with humans observed on 525 nights for 1239 (group × night) observations

	ne Botswana^a^		QENP^b^
Den type	Syn^c^	Apo^d^	
Man-made structures			
Buildings and structures	38		3
Building material	20		
Scrap	15		
French drains	7		
Overturned boat	1		
Slash pile	1		
Total	81	0	3
Natural structures			
Hollow logs	1	67	
Termite mounds	6	28	65
Holes in trees	3	6	
Hole in ground	2		11
Rocks	6		
Erosion gullies			21
Total	19	100	97
Number of observations	1203	36	144
Number of groups	11	6	6

^a^Northeastern Botswana (Chobe National Park, Kasane Forest

Reserve, towns of Kasane and Kazungula) (this study)

^b^Queen Elizabeth National Park, Uganda [45]

^c^Synanthropic (living in association with humans)

^d^Apoanthropic (living without association with humans)

### Effects of experimental removal of anthropogenic food resources

As a natural experimental confirmation of observational results for the effects of living in association with humans, a group that lived in association with humans (ID = 1) with access to only 1 lodge refuse site during the study period, expanded its dry season space use when that refuse site was closed. This group was the largest in our study but had the second smallest home range, concentrated around a lodge in Chobe National Park. Relative to our first dry season data (2008), this group exhibited only moderate changes in space use over the next 2 study years (2009 and 2010). Dry season home range increased 0.4-fold each year; dry season cores increased 1-fold and 0.3-fold, respectively. Between 2010 and 2011 dry seasons this lodge’s refuse site was closed. In 2011 this group increased dry season range 2-fold, and core range 3-fold (Table 2, prediction 6), putatively in response to losing the refuse site, a rich concentrated anthropogenic food resource. Over the 2009, 2010, and 2011 dry seasons, none of the other study groups living in association with humans were excluded from their refuse sites and the median space use metrics of all groups remained relatively constant compared to the 2008 data (median dry season home range increased 0.3-fold, 0.1-fold, and 0.1-fold, respectively; median dry season cores increased 0.8-fold, 0.2-fold, and decreased 0.03-fold, respectively).

### Home range metrics

Groups exhibited yearly site fidelity, barring 1 GPS-collared group in 1 year. This group exhibited a long-distance dispersal in that year, and we removed the associated data from our analyses. We also excluded 1 home range that did not approach an asymptote. Home range sizes approached asymptotes at a median of 335 fixes (IQR: 135 to 478). Time to statistical independence was 4 h for 6 groups combined. The median 95% kernel density home range for study mongooses was 68 ha (IQR: 39 to 134) and median core range was 15 ha (IQR: 9 to 28), with core ranges delineated at non-arbitrary volume contours (median: 66%; IQR: 58 to 71) (*n*=10 groups with and without human association; Table 4). The median home range for the groups that lived in association with humans was 46 ha (IQR: 37 to 98, *n*=8), with a median core range of 11 ha (IQR: 9 to 14, *n*=8). The 2 groups that lived without association with humans had home ranges of 131 ha and 194 ha, with cores of 74 ha and 50 ha. The median wet and dry season ranges for 7 groups that lived in association with humans were 40 ha and 27 ha, respectively. For the group living without association with humans for which we had adequate seasonal data, the wet and dry season ranges were 94 ha, and 124 ha, respectively. Median wet and dry season day ranges for 6 groups that lived in association with humans were 1.5 km and 0.9 km, respectively (Table 4).

**Table 4:** Home ranges, overall core ranges, seasonal home ranges, seasonal core ranges, and day ranges for 10 banded mongoose groups in northeastern Botswana (2008 – 2011)

Group		Overall home range (ha)					Seasonal range (ha)				Day^a^ (km)	
							Wet (ha)		Dry (ha)		Wet	Dry
ID	PC1^b^	*n*_*locs*_^c^	Asym.^d^	95%	Core	%^e^	95%	Core	95%	Core		
2	-2.36	206	62	131	74	77	94	43	124	50		
10	-2.18	80	70	194	50	61						
13	-0.92	79	66	175	31	55						
3	0.00	843	500	50	9	71	38	11	31	11	1.7	0.6
1	0.28	1159	900	33	9	70	28	9	12	6	1.3	0.9
11	0.45	397	330	41	12	67	47	21	27	12	2.2	0.9
6	0.83	585	340	38	7	55	40	13	26	7		
5	1.01	2284	1250	134	17	65	164	30	68	22	2.1	0.9
8	1.22	867	330	86	18	57	73	16	38	7	0.4	0.2
9	1.66	593	410	26	7	75	23	6	22	7	0.4	1.2

^a^Day range (daily distance traveled)

^b^Principal Component 1 (level of association with humans: ordered from least association to most association)

^c^Number of relocations

^d^Asymptote: Number of relocations at which estimates approached an asymptote

^e^Percent volume contour for statistical core range

### Effects of seasonality

**Fig. 4:**
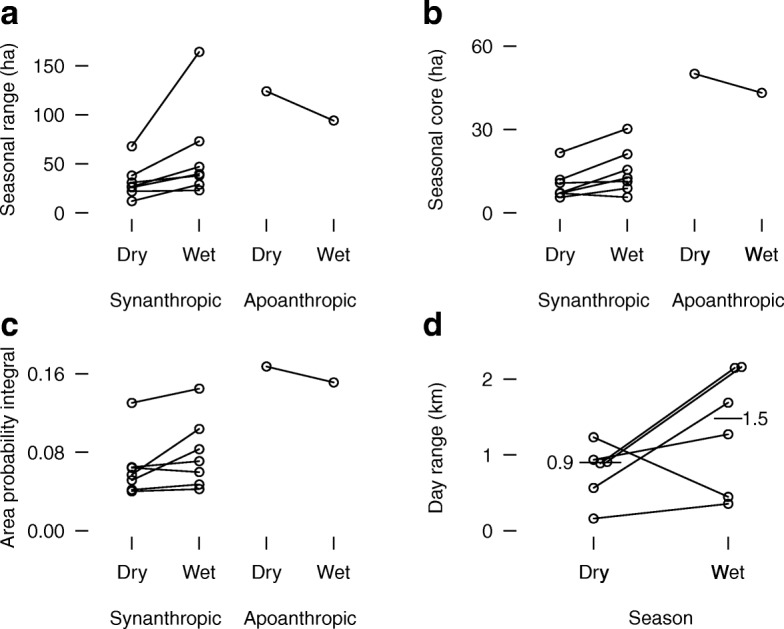
Seasonal home range (**a**), seasonal core range (**b**), and seasonal space use dispersion (area-probability integral) (**c**) in 7 groups of banded mongooses living in association with humans and 1 group lacking association with humans in northeastern Botswana (2008 – 2011). Seasonal day range (**d**) for 7 groups living in association with humans. Seasonal values within a group are connected by lines

Contrary to seasonal metabolic predictions (Table 2, prediction 3), space use among groups living in association with humans was more dispersed in the wet season than in the dry season. Seasonal home ranges (with 49:1 odds, Fig. 4a), seasonal core ranges (with 49:1 odds, Fig. 4b), space use dispersion (with 16:1 odds, Fig. 4c), and day ranges (with 10:1 odds, Fig. 4d) were all greater in the wet season than in the dry season, with effect sizes of 18.4 ha (0.5-fold relative increase), 4.9 ha (0.6-fold relative increase), 0.014 (0.1-fold relative increase), and 606 m (1.3-fold relative increase), respectively (Table 5). Seasonal space use relationships for the group that lacked association with humans matched a priori predictions (Table 2, prediction 3).

**Table 5:** Bayesian parameter estimation, testing for paired seasonal differences (dry season minus wet season) in space use, group size and habitat metrics within banded mongoose groups in northeastern Botswana (2008 – 2011)

Response variable	Pred. ^a^	n^b^	*β* ^c^	95% HPDI^d^		% Posterior^e^		Result^f^	Interp.^g^
				LB	UB	< 0	> 0		
Groups living in association with humans									
Day range (km)	D > W	6	-0.6	-1.6	0.4	91	9	D < W	MC
Seasonal home range (ha)	D > W	7	-18.4	-37.0	-0.3	98	2	D < W	HC
Seasonal core range (ha)	D > W	7	-4.9	-9.4	-0.5	98	2	D < W	HC
API^h^	D > W	7	-0.014	-0.033	0.005	94	6	D < W	MC
Building density (ha ^−1^)		7	0.49	0.04	0.92	2	98	D > W	HC
Macrofauna density (m ^−2^)	D < W	7	-301	-640	8	97	3	D < W	HC
Tourist bed nights^i^			0.23	0.04	0.43	1.2	98.8	D > W	HC
Fecal organic matter (%)	D = W	7	-1	-11	9	60	40	D < W	U
Group size	D < W	9	7.3	0.9	13.6	1	99	D > W	HC
Groups living without association with humans									
Seasonal home range (ha)	D > W	1	30					D > W	U
Seasonal core range (ha)	D > W	1	7					D > W	U
API^h^	D > W	1	0.016					D > W	U
Macrofauna density (m ^−2^)	D < W	1	-432					D < W	U
Fecal organic matter (%)	D = W	1	-2					D < W	U
Group size	D < W	1	8					D > W	U

^a^Prediction of relationship based on theory: dry season (D), wet season (W)

^b^Number of paired seasonal estimates (i.e. mongoose groups) used

^c^Effect size

^d^Highest posterior density interval (HPDI) with lower bound (LB) and upper bound (UB)

^e^Percentage of posterior distribution below or above zero

^f^Result: dry season (D), wet season (W)

^g^Revised interpretation of the result (posterior): moderate certainty (MC), high certainty (HC), uncertainty (U)

^h^Dispersion of space use — area-probability integral (API)

^i^Dry season minus wet season bed nights sold, expressed as relative (X-fold) change, for 10 tourist lodges

Within a group, seasonal differences in fecal organic matter content were negligible and uncertain for groups living in association with humans and for the group living without association with humans (Table 5). Yet, seasonal macrofauna density for the group living without association with humans matched those for groups living in association with humans and matched the seasonality prediction that soil macrofauna would occur at lower densities in the dry season than in the wet season (with 32:1 odds for the groups living in association with humans) (Table 5).

### Effects of resource richness versus dispersion

Among all groups (those living with or without association with humans combined), there was strong evidence that resource richness factors and not resource dispersion or group size were related to variation in space use dispersion (Tables 6 and 7). Space use dispersion (the area-probability integral) was negatively related to building count (moderate certainty, 9:1 odds for a negative relationship) and building density (high certainty, 49:1 odds for a negative relationship) (Table 2, prediction 5). Neither patch dispersion (Table 2, prediction 9), nor group size (Table 2, predictions 2 and 8) were related to dispersion of space use in either season (evidence was weak i.e. the outcome was uncertain, Table 6). There was, however, some suggestion that patch dispersion could explain variation in the variation in seasonal home ranges, but only during the wet season (Table 7). Contrary to our prediction of a negative relationship, variation in seasonal home range and core range size appeared to be positively-associated with soil macrofauna density, during both seasons (Table 7).

**Table 6:** Bayesian simple linear regression analyses of banded mongoose (northeastern Botswana, 2008 – 2011) responses in seasonal concentration of space use (area-probability integral) to factors putatively associated with association with humans, metabolic scaling theory, and the resource dispersion hypothesis

Response variable	Pred.^a^	n^b^	*β* ^c^	95% HPDI^d^		% Post.^e^		DIC^f^	Interp.^g^
Predictor				LB	UB	< 0	> 0		
Area-probability integral (dispersion of space use)									
Dry season									
Buildings^h^	-	9	−2.3×10^−4^	−6.2×10^−4^	1.5×10^−4^	90	10	-25.6	MC
Buildings (core)^h^	-	9	−1.4×10^−3^	−3.4×10^−3^	6.1×10^−4^	93	7	-26.4	MC
Building density^h^	-	9	−2.1×10^−2^	−4.1×10^−2^	−1.5×10^−3^	98	2	-29.9	HC
Building density (core)^h^	-	9	−1.4×10^−2^	−2.9×10^−2^	2.1×10^−3^	96	4	-28.1	HC
Macrofauna^i^	-	9	−5.2×10^−10^	−1.4×10^−8^	1.1×10^−8^	60	40	18.2	U
Macrofauna (core)^i^	-	9	1.7×10^−8^	−1.5×10^−7^	1.8×10^−7^	21	79	79.9	U
Macrofauna density^i^	-	9	−2.4×10^−8^	−1.2×10^−6^	6.0×10^−7^	44	56	31.7	U
Macrofauna dens. (core)^i^	-	9	−5.2×10^−7^	−4.3×10^−6^	1.3×10^−6^	59	41	-17.7	U
Patch dispersion	+	9	−9.2×10^−3^	−2.9×10^−2^	9.9×10^−3^	86	14	-24.8	U
Group size	+	9	−4.4×10^−4^	−3.7×10^−3^	2.9×10^−3^	62	38	-23.1	U
Wet season									
Buildings^h^	-	8	−1.3×10^−4^	−4.5×10^−4^	1.9×10^−4^	84	16	-23.9	U
Buildings (core)^h^	-	8	−1.9×10^−4^	−2.7×10^−3^	2.3×10^−3^	57	43	-22.3	U
Building density^h^	-	8	−2.2×10^−2^	−7.1×10^−2^	2.7×10^−2^	85	15	-24.0	U
Building density (core)^h^	-	8	−1.5×10^−2^	−5.5×10^−2^	2.4×10^−2^	81	19	-23.6	U
Macrofauna^i^	-	8	−2.8×10^−10^	−6.1×10^−9^	1.0×10^−8^	57	43	31.0	U
Macrofauna (core)^i^	-	8	5.3×10^−9^	−6.0×10^−8^	6.4×10^−8^	32	68	49.6	U
Macrofauna density^i^	-	8	−2.5×10^−8^	−1.7×10^−6^	1.1×10^−6^	54	46	61.2	U
Macrofauna dens. (core)^i^	-	8	3.9×10^−8^	−4.8×10^−7^	6.0×10^−7^	37	63	49.8	U
Patch dispersion	+	8	−2.9×10^−3^	−2.3×10^−2^	1.7×10^−2^	64	36	-22.4	U
Group size	+	8	−1.2×10^−3^	−6.9×10^−3^	4.6×10^−3^	69	31	-22.7	U

^a^Prediction of relationship based on theory

^b^Number of mongoose groups used

^c^Effect size

^d^Highest posterior density interval (HPDI) with lower bound (LB) and upper bound (UB)

^e^Percentage of posterior distribution below or above zero

^f^Deviance information criterion

^g^Revised interpretation of the result (posterior): moderate certainty (MC), high certainty (HC), uncertainty (U)

^h^Number of buildings in home range or density (buildings ha ^−1^)

^i^Number of macrofauna items theoretically available in home range or density (macrofauna m ^−2^)

**Table 7:** Bayesian simple linear regression analyses of banded mongoose (northeastern Botswana, 2008 – 2011) responses in seasonal home ranges and seasonal core ranges to factors putatively associated with association with humans, metabolic scaling theory, and the resource dispersion hypothesis

Response variable	Pred.^a^	n^b^	*β* ^c^	95% HPDI^d^		% Post.^e^		DIC^f^	Interp.^g^
Predictor				LB	UB	< 0	> 0		
Seasonal home range size									
Dry season									
Building density^h^	-	9	-23.0	-49.4	3.1	96	4	102.9	HC
Building density (core)^h^	-	9	-19.1	-36.4	-1.7	98	2	101.2	HC
Macrofauna density^i^	-	9	1.7×10^−5^	2.8×10^−6^	3.0×10^−5^	1	99	294.4	HC
Macrofauna density (core)^i^	-	9	2.3×10^−5^	8.6×10^−6^	3.7×10^−5^	1	99	294.4	HC
Patch dispersion	+	9	-11.8	-34.2	10.1	87	13	105.0	U
Group size	+	9	-1.0	-4.7	2.8	72	28	106.2	U
Wet season									
Building density^h^	-	9	12.8	-34.2	59.6	27	73	88.8	U
Building density (core)^h^	-	9	-28.3	-55.7	-0.7	98	2	84.3	HC
Macrofauna density^i^	-	9	7.1×10^−6^	5.1×10^−6^	9.0×10^−6^	1	99	1319.6	HC
Macrofauna density (core)^i^	-	9	1.04×10^−5^	1.002×10^−5^	1.1×10^−5^	1	99	-75213.5	HC
Patch dispersion	+	9	-10.0	-25.2	5.3	92	8	87	MC
Group size	+	9	0.005	-5.1	5.2	50	50	89.2	U
Seasonal core range size									
Dry season									
Building density^h^	-	9	-7.2	-12.3	-2.2	99	1	78.7	HC
Macrofauna density^i^	-	9	7.7×10^−6^	2.9×10^−6^	1.2×10^−5^	1	99	-4316.3	HC
Patch dispersion	+	9	-2.1	-8.9	4.4	76	24	86.1	U
Group size	+	9	-0.2	-1.4	1.0	63	37	86.5	U
Wet season									
Building density^h^	-	9	-10.6	-14.2	-6.9	99	1	50.6	HC
Macrofauna density^i^	-	9	3.1×10^−6^	1.6×10^−6^	4.7×10^−6^	1	99	1208.9	HC
Patch dispersion	+	9	-3.2	-6.4	-0.02	98	2	63.3	HC
Group size	+	9	0.1	-1.3	1.5	45	55	68.1	U

^a^Prediction of relationship based on theory

^b^Number of mongoose groups used

^c^Effect size

^d^Highest posterior density interval (HPDI) with lower bound (LB) and upper bound (UB)

^e^Percentage of posterior distribution below or above zero

^f^Deviance information criterion

^g^Revised interpretation of the result (posterior): moderate certainty (MC), high certainty (HC), uncertainty (U)

^h^Number of buildings in home range or density (buildings ha ^−1^)

^i^Number of macrofauna items theoretically available in home range or density (macrofauna m ^−2^)

## Discussion

Our study shows 3 primary results: 1) that banded mongoose space use is strongly affected by a group’s level of association with humans, 2) that banded mongoose space use provides support for predictions from the metabolic theory of ecology, but only when seasonality and association with humans, and their interaction, are explicitly factored in, and 3) that banded mongoose space use provides minimal support for predictions from the resource dispersion hypothesis (Table 2).

Evidence from 1 group that lacked association with humans matched seasonal metabolic theory predictions, that animals should range farther to meet their metabolic requirements during seasons when food resources — e.g. macrofauna — are less available. Yet, association with anthropogenic resources reversed historically-expected outcomes for groups living in association with humans. During dry seasons, groups living in association with humans had smaller ranges, core ranges, day ranges, increased concentration of space use, and used areas of higher building density, than during wet seasons. Fecal organic matter levels may provide an indication of food limitation. If animals are meeting their metabolic requirements through behavioral plasticity, then fecal organic matter levels should remain consistent across putatively food-limiting seasons. Fecal organic matter levels within groups differed between the wet and dry seasons by only 1% and 2% for groups living in association with humans and a group living without association with humans, respectively. Yet, the group living without association with humans had more dispersed space use during the dry season than the wet season and the groups living in association with humans had more concentrated space use during the dry season than the wet season, while soil macrofauna densities were lower during the dry season than the wet season for all mongoose groups. Further, overall fecal organic matter levels were higher in groups with greater levels of association with humans, yet, estimated soil macrofauna densities showed no clear relationship with level of association with humans. Anthropogenic food sources may thus partially replace soil macrofauna in the dry season diet of mongoose groups living in association with humans, coincident during this period with lower soil macrofauna density, more tourism and hence, more food waste, driving the seasonality-synanthropy interaction.

Resource richness (anthropogenic or soil macrofauna) was associated with dispersion of space use during the dry season, a putatively resource-poor time period when soil macrofauna are less available or less abundant. Group size and patch dispersion had no apparent relationship to space use dispersion or range size in the dry season. This lack of relationship between group size and range size has also been demonstrated in banded mongooses in Uganda [28], in accordance with 1 prediction of the resource dispersion hypothesis, but this finding constitutes only weak support for the resource dispersion hypothesis overall. Thus, data for banded mongooses support predictions for synanthropic metabolic theory and seasonal metabolic theory, but only weakly 1 prediction from the resource dispersion hypothesis. This suggests a seasonal synanthropic metabolic theory for banded mongoose space use ecology, in which resource richness factors, which include anthropogenic food resources, drive space use in the food-limited season — with more human food resources, mongooses require less space to meet their energetic requirements. These results could improve understanding of wildlife responses to increasingly human-modified landscapes.

While it is difficult to compare among studies due to methodological differences, home ranges calculated in this study were, overall, similar to those reported in Uganda. Population density in our study area, however, was considerably lower than in Uganda, and the space use for groups living in association with humans was considerably more concentrated (Table 1). The variability in population density across the geographic range of the species (Table 1) and what this means for space use, home range overlap, and static and dynamic interaction among groups are areas ripe for further investigation.

Our results regarding effects of living in association with humans on space use echo those for banded mongooses from Uganda, where refuse-feeding groups with richer resources had smaller core ranges than other groups [28]. Yet, our approach differs by using statistical core ranges, by using area-probability integrals, a metric for space use concentration, and by using groups along a gradient of anthropogenic landscape change. Combined, the studies provide support for synanthropic metabolic theory in banded mongooses — more abundant food resources in the form of anthropogenic resources allow banded mongoose groups to meet their energetic requirements in less space. Our synanthropic metabolic theory findings were similar to those for other carnivorans, including golden jackals (*Canis aureus*) [26], and Eurasian badgers (*Meles meles*) [77]. In species where populations living in association with humans have larger home ranges than populations living without human association, as in bobcats (*Lynx rufus*) [78] and coyotes (*Canis latrans*) [27, 78], increased persecution by humans and differences in anthropogenic food waste management may overwhelm synanthropic metabolic effects. Coyotes may demonstrate this combination of persecution effects and synanthropic metabolic effects, by exhibiting smaller home ranges at refuse sites [29], and larger home ranges in urban areas [27, 78] where persecution is putatively higher.

Contrary to metabolic theory predictions, total macrofauna and macrofauna density were positively related to space use dispersion. This warrants further investigation. The effect of anthropogenic food resources in this system may have reversed the expected relationship, by effectively substituting macrofauna in the diet of groups living in association with humans. Perhaps resource richness should be estimated within core ranges (i.e. the areas within a home range where space use is concentrated), rather than overall home ranges, where utilization distribution tails may contribute disproportionately to resource estimates (i.e. the areas surrounding the cores where space use is distributed randomly or evenly may contribute disproportionately to our calculation of total macrofauna, even though these areas were used relatively infrequently). Our analyses that did measure resources in the core ranges yielded similar results to our analyses using resource measurements made across a mongoose home range (Tables 6 and 7).

The resource dispersion hypothesis does not fit with banded mongoose space use, as it does with space use by large carnivores, such as African lions (*Panthera leo*) [79]. This contrast may suggest species-specific differences, but other factors could explain lack of support for the hypothesis. Banded mongooses exhibit obligate group-living and their social behavior may mask resource dispersion predictions, or we may have modeled resource richness or dispersion inappropriately [80]. Characterizing a foraging patch can be problematic, especially when one has to combine foraging on natural food resources with foraging on anthropogenic resources such as a large refuse site. Unfortunately, with scarce but large “bonanza” resources, such as refuse sites, resource dispersion and resource richness become conflated.

Future studies might assess dispersion of macrofauna patches, buildings, and refuse sites separately, and assess longitudinal change in space use dispersion, using area-probability integrals, rather than home and core ranges. In general, while we measured proxies for banded mongoose resources in our study, future studies should aim to more directly measure factors such as anthropogenic food richness or availability. It will also be intriguing to consider how these anthropogenic resources may also include evolutionarily-novel costs. While food and denning resources are obvious benefits to banded mongooses, space use will also reflect trade-offs with possible costs such as predation risk from domestic dogs, disease transmission e.g. [81], persecution from humans, and road mortality.

While we have shown evidence suggesting that variability in food resource richness, resulting from seasonality and anthropogenic resources, may be a possible mechanism driving variation in banded mongoose space use, predation risk could also be important. Studies comparing the relative importance of food resource availability and predation risk in driving space use have yielded mixed results. In some species, such as Arctic ground squirrels (*Spermophilus parryii*) [82], and female oribi (*Ourebia ourebi*) [83], food resource availability appears to be more important, while in other species, such as vervet monkeys (*Cercopithecus aethiops*) [84] and samango monkeys (*Cercopithecus mitus erythrarcus*) [85], predation risk appears to be more important. Future studies should assess predation risk as a possible mechanism driving banded mongoose space use. In our study area, association with humans could have resulted in the reduction or exclusion of free-living carnivoran predators that typically prey upon banded mongooses, but association with humans may also have resulted in increases in domestic dogs which we recorded preying upon mongooses. It is unclear whether association with humans would have affected avian predator densities or behavior in our study area, and, overall, it is unclear how association with humans may have altered predation risk for banded mongooses.

Predictions that could be tested in the future include: 1) mongooses exhibit a higher propensity to forage under canopy cover than in the open within the same habitat; 2) buildings and refuse sites represent patches for banded mongooses foraging on human food waste and hence, mongooses exhibit a higher propensity to forage at these anthropogenic resources than away from them in an urban environment. These two predictions could be tested using the Marginal Value Theorem and giving-up densities. An additional prediction for future testing could include: 3) spatial and temporal variability in predation risk will, similar to heterogeneous food resources, be associated with variability in banded mongoose space use — mongooses will exhibit dispersion of space use in areas with high perceived predation risk and will concentrate space use in areas with low perceived predation risk. We think that anthropogenic food resources and urban-associated predation or mortality risks are both relatively recent cues and thus potentially evolutionarily-novel for banded mongooses. Further, we think that the predation risk cues associated with urban environments may take more generations than the anthropogenic food cues for mongooses to develop appropriate cue recognition and response systems. Thus, we predict that 4) in urban environments, variability in anthropogenic food resources will explain more of the variability in mongoose space use than will variability in urban-associated predation risk.

## Conclusions

Human-modified environments have large effects on banded mongoose space use, highlighting the important implications for landscape change on wildlife behavior [7]. As with many other species [6], much of the previous research on banded mongooses has been conducted at sites with relatively low anthropogenic impact, often in national parks and nature reserves. Ours is the first banded mongoose study to include contiguous groups living in a national park, as well as those living at tourist lodges, in the park or in nearby forest reserves, and groups living in an urban landscape. Our results indicate fundamental differences in the space use and behavior of banded mongooses between groups living with or without association with humans. This suggests that management and conservation of banded mongooses could take this classification dichotomy into account, if not a gradient of human association.

Greater consideration of the effects of anthropogenic resources on organisms living in association with humans may be useful for improving our understanding of basic ecology, and for advancing our theoretical frameworks. As humankind’s footprint continues to expand and affect a greater proportion of natural environments and the organisms they support, this issue will become increasingly important in the management and conservation of biodiversity, possibly suggesting dual approaches for populations living with or without association with humans in a range of species. A more nuanced appreciation of organismal ecology in anthropogenically-modified landscapes will also allow us to more effectively achieve the goals of reconciliation ecology: to design urban spaces with compatibility with biodiversity in mind [86].

## Abbreviations

API: Area-probability integral
Apo: Apoanthropic
Asym: Asymptote
CNP: Chobe National Park
D: Dry season
DIC: Deviance information criterion
GPS: Global positioning system
HC: High certainty
HNP: Hwange National Park
HPDI: Highest posterior density interval
Interp: Interpretation
IQR: Interquartile range
KDE: Kernel density estimation
KNP: Kruger National Park
LB: Lower bound
LSCV: Least-squares cross-validation
MC: Moderate certainty
MCMC: Markov Chain Monte Carlo
MCP: Minimum convex polygon
MPNR: Mosdene Private Nature Reserve
PC1: First principal component
Pred: Prediction
QENP: Queen Elizabeth National Park
SNP: Serengeti National Park
Syn: Synanthropic
U: Uncertainty
UB: Upper bound
VCNR: Vernon Crookes Nature Reserve
VHF: Very high frequency
W: Wet season
